# Suitable Granular Road Base from Reclaimed Asphalt Pavement

**DOI:** 10.3390/ma18040854

**Published:** 2025-02-15

**Authors:** Oswaldo Guerrero-Bustamante, Amparo Guillen, Fernando Moreno-Navarro, M. C. Rubio-Gámez, Miguel Sol-Sánchez

**Affiliations:** 1 Construction Engineering Laboratory, University of Granada (LabIC.UGR), 18071 Granada, Spain; oguerrer5@cuc.edu.co (O.G.-B.); amguillen@ugr.es (A.G.); fmoreno@ugr.es (F.M.-N.); mcrubio@ugr.es (M.C.R.-G.); 2 Department of Civil & Environmental, Universidad de la Costa, Barranquilla 080003, Colombia

**Keywords:** granular base, Reclaimed Asphalt Pavement (RAP), sustainable materials, road infrastructure

## Abstract

The granular bases commonly used in the construction of road infrastructure projects often require a high consumption of raw materials. The potential utilization of recycled materials, specifically Reclaimed Asphalt Pavement (RAP) derived from road asphalt pavement demolition, emerges as a promising sustainable advantage for infrastructure projects, considering its potential environmental and cost benefits in other layers of the structure. In this context, this research proposes a feasibility study on the use of RAP as a granular base layer, supported by an advanced laboratory analysis that includes a range of tests simulating the in-service conditions as well as a full-scale demonstration of the material behavior under static and dynamic loads. Various design variables, such as different gradations and binder content, are considered. The results demonstrate that, despite having discontinuous gradation and smaller aggregate sizes than those commonly applied in natural base layers, the evaluated recycled materials exhibit a higher load-bearing capacity and resistance to permanent deformation than the reference materials commonly used as granular bases. Notable enhancements of up to 30% in elastic modulus, coupled with reductions of around 20% in permanent deformations, have been documented using the asphalt cement potential in the old pavement.

## 1. Introduction

The construction and maintenance of road infrastructures represent a constant challenge in the field of civil engineering and sustainable development. In this context, the search for alternatives that not only meet technical performance standards but also address environmental and economic concerns has become a strategic priority [1,2]. Reutilizing waste materials has emerged as a viable alternative for road maintenance, rehabilitation, and even in the construction of new pavements [3]. One of the most promising approaches is the use of Reclaimed Asphalt Pavement (RAP), as a key component in the structural layers of pavement [4].

The global demand for aggregates in the construction sector is estimated to be between 20 and 40 billion tons annually. This demand is projected to grow at an annual rate of about 6.8% until 2031 [5,6], positioning the construction sector as one of the most environmentally impactful industries [7]. The sector is associated with 40% of global greenhouse gas emissions and accounts for 30% of worldwide water consumption [8,9,10]. The generation of demolition waste worldwide remains a significant problem. In Australia, approximately 1.2 million tons of RAP are produced annually [11], while in the United States, construction waste production reaches 30 million tons [12]. In many cases, only 40% of this waste is reused in the construction sector [13].

While RAP is primarily reused in asphalt mixtures [14,15,16], its incorporation is often limited to approximately 20–30% due to logistical constraints and regulatory restrictions [17]. This restricted use highlights the need to explore alternative applications for RAP to maximize its potential in infrastructure projects. One such application is its use in granular base layers, which play a fundamental role in the stability and durability of pavement structures. Traditionally, these layers have been constructed using natural or virgin aggregates, requiring extensive resource extraction and raising environmental concerns. In this context, RAP emerges as a viable and sustainable alternative.

A report from the Virginia Center for Transportation Innovation and Research [18] demonstrated the technical feasibility of using RAP in unbound base layers, a practice already adopted by numerous agencies in the United States. However, there is a lack of technical literature compiling the current field performance of these applications, highlighting the need for further research into the mechanical behavior and durability of RAP-based granular layers under real-world service conditions.

RAP has appropriate characteristics that make it appealing in terms of sustainability [4,19]. Its composition, enriched with residual asphalt, can contribute to the load-bearing capacity and strength of the material [20]. However, some studies have indicated reductions in CBR strength [21] and compacted density of the materials when RAP is included [21,22]. Conversely, increases in the resilient modulus have been observed [22]. Previous experience has shown the effective utilization of reclaimed asphalt pavement (RAP) in granular layer applications. Recent research highlights the testing of mixes incorporating 50% and 75% RAP alongside fresh granular materials and recycled concrete aggregate to evaluate their suitability for granular base layers [3,23]. The incorporation of RAP in the construction of granular base and sub-base layers not only imparts structural advantages but also leads to cost savings and environmental benefits by diminishing reliance on natural aggregates and promoting sustainability [24,25,26]. Numerous studies have indicated that granular layers incorporating RAP can deliver strength equal to or surpassing that of traditional base and sub-base layers, thereby contributing to economically viable and sustainable road construction practices [27].

Despite its potential, the mechanical properties of RAP-based granular layers remain insufficiently studied [3,28], particularly under realistic service conditions. The heterogeneity of RAP due to variations in aggregate composition, asphalt content, and processing methods necessitates further investigation. Advanced studies, including full-scale evaluations, are required to assess the mechanical behavior of RAP-based layers comprehensively and ensure their reliable implementation in infrastructure projects.

In general, to fully power these advantages, it is essential to thoroughly understand its mechanical behavior, its interaction with other pavement layers, and its response to specific design variables, considering that this material from the crushing or milling of asphalt pavements is highly heterogeneous and possesses unique mechanical properties. Thus, the primary purpose of this research is to explore the technical feasibility of using RAP from the demolition of deteriorated pavements as a granular base layer in new pavement structures under expected service conditions, supported by an advanced laboratory study that includes the incorporation of RAP with different physical properties. The research includes physical-mechanical characterization assessing different variables while carrying out full-scale testing box evaluation of the material mechanical behavior in terms of load-bearing capacity and resistance to permanent deformations, considering the current limited number of studies thoroughly investigating this application under real service conditions.

## 2. Materials and Methods

### 2.1. Materials

In this study, four materials were employed: one natural reference aggregate and three RAP samples with different characteristics to analyze the impact of their property variability (e.g., source and processing) on the feasibility assessment. The natural reference aggregate corresponds to a type of crushed stone of limestone origin, commonly used as a granular base in road infrastructure in Spain. This material exhibits a continuous particle size distribution with particle fractions in the range of 0.0/32.0 mm. Moreover, the granular material exhibited suitable physical and mechanical properties for its application as a granular base, adhering to Spanish standards [29].

The three types of RAP (referred to as RAP 1, RAP 2, and RAP 3) were collected from different roadways in Andalusia (Spain), then processed at a plant, crushed, and classified into 0/22 mm fractions to ensure uniformity and obtain a more representative sample for use in this study. These materials exhibit different particle size distributions and binder content, which makes them appropriate for use in this study, which analyzes the influence of RAP variability. The main properties are listed in Table 1. It is noted that the recycled materials have varying amounts of residual binder content, ranging from 3.0% to 4.0% of the total weight.

**Figure 1:**
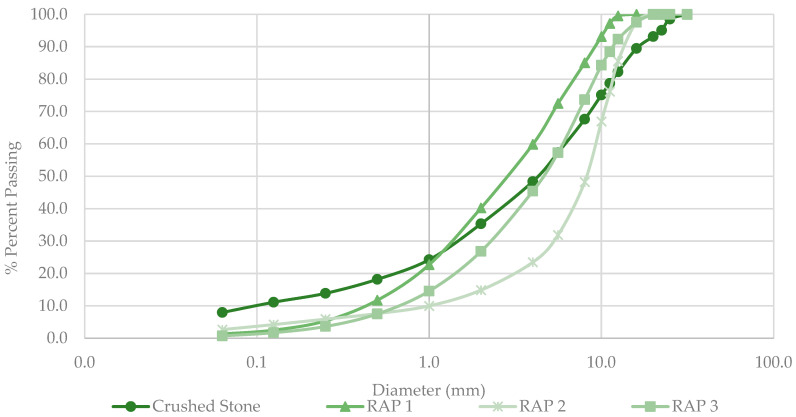
Particle size distribution.

Similarly, it can be observed in Figure 1 that there are RAP materials with different particle size distributions; RAP 1 consists of fine particles within the particle size range of 0/16.0 mm, whereas RAP 2 is characterized by coarser particles, falling within the range of 0.0 to 20.0 mm. Lastly, RAP 3 is categorized as an intermediate material, with particle sizes ranging from 0.0 to 20.0 mm.

### 2.2. Testing Plan

The testing plan conducted in this research is presented in Table 2; it consists of five main stages: the first one consists of the (i) study of the mechanical behavior and parametric analysis of designing variables for the RAP base layer in order to study the effect of the RAP type and RAP replacement on natural aggregates in different proportions; subsequently, for the materials exhibiting optimal performance in this initial stage, (ii) bearing capacity using a 300 mm load plate, and (iii) resistance to permanent deformation tests were conducted in a 35 cm × 35 cm test box, aiming to obtain preliminary information on the mechanical behavior of these materials. In the same direction, (iv) the ability to protect the lower layers was tested [40], and (v) a full-scale laboratory testing box to analyze the behavior under real operating conditions.

To study the mechanical behavior of the recycled materials and the crushed stone (reference), this research examines the effects of different design variables in the use of RAP for the base layer, analyzing their influence on crucial mechanical properties as follows:

- Influence of RAP type: To achieve this goal, cylindrical specimens were manufactured and compacted with the optimal moisture content determined in the characterization phase. For specimens containing recycled materials, re-compaction was performed 24 h later at a temperature of 60 °C, considering the presence of a thin layer of adhered bitumen that enhances the mechanical behavior of the material [41] and that this temperature can be reached on-site [42,43,44]. A direct comparison was made between the reference materials and the recycled materials.
- Impact of combining RAP with reference materials: In this part of the study, the effect of replacing RAP in the crushed stone on mechanical behavior was evaluated with the aim of assessing the viability of partial replacement of virgin material with recycled material, in this case, in a 25/75, 50/50 and 75/25 recycled/virgin material ratio based on previous studies [45,46,47].

In this case, the tests proposed to evaluate the mechanical behavior are simple compression strength and indirect tensile strength; they were selected based on their suitability for assessing granular materials. The simple compression test is particularly appropriate for unbound granular materials, providing insights into their load-bearing capacity under compressive loads. Meanwhile, the indirect tensile strength test was chosen to investigate whether the presence of adhered bitumen in RAP materials contributes to tensile resistance, potentially enhancing the cohesion of the mixture [48].

For the simple compression test, cylindrical specimens with a diameter of 100 mm and a height of 200 mm were used, using granular materials (reference and RAPs) without any binder and compacted with the gyratory compactor according to standard UNE 103400:1993 [49]. The indirect tensile strength tests were conducted on cylindrical samples with a diameter of 100 mm and a height of 60 mm based on standard UNE-EN 12697-23:2018 [50]. To ensure the reliability of the results, three specimens were prepared for each test. As mentioned before, the cylindrical specimens were manufactured with the optimal moisture content found in the material characterization phase. Additionally, for specimens that included recycled materials (RAPs), were compacted again 24 h later at a temperature of 60 °C, aiming to take advantage of the agglomeration capacity provided by the residual bitumen.

For the recycled material with better performance (Opt. RAP) and the reference material, a load-bearing test was conducted using the 300 mm static and dynamic plate load test according to standards UNE 103808:2006 [51] and UNE 103807-2 [52], respectively. To ensure the reliability of the results, three test boxes were prepared for each condition. For the static load test, two loading cycles were performed, each with six steps with approximately equal increments (85 kPa, 200 kPa, 300 kPa, 400 kPa, 500 kPa, and 600 kPa). The dynamic plate load test, on the other hand, was conducted by applying loading cycles with an amplitude of 420 kPa at a frequency of 5 Hz. Both tests allow for determining the vertical modulus of elasticity under static load (E_V_) and dynamic load (E_DYN5HZ_) for each of the studied materials.

On these same materials, permanent deformation or rutting tests were conducted using the Wheel Tracking device using two specimens for each condition according to the UNE-EN 12697-22 standard [53]. In this case, the material’s susceptibility to deformation is evaluated by measuring the rut depth produced by repeated passes at a fixed temperature of 25 °C. A wheel is subjected to a standardized load of 700 N with a total travel of 230 mm at an approximate frequency of 26 cycles per minute.

**Figure 2:**
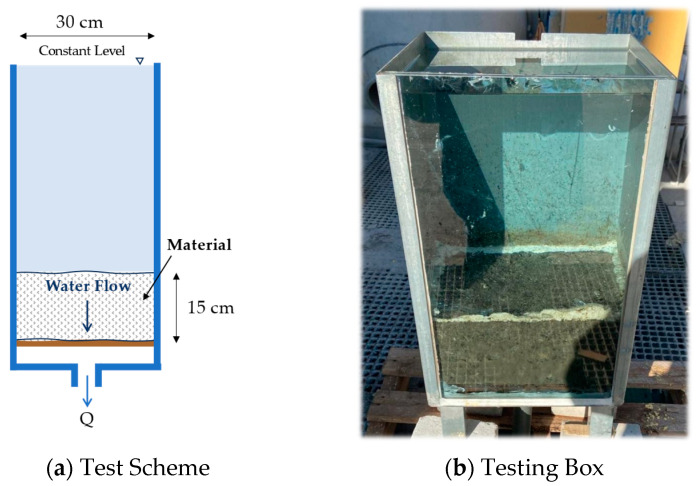
Permeability test setup.

A constant head permeability test adapted from standard UNE-EN 103403:1999 [54] was conducted using a 300 mm × 300 mm box with a height of 500 mm, as can be seen in Figure 2. For each material, three tests were performed. The coefficient of permeability was calculated for the studied materials, defined as the water flow through a unit cross-sectional area under a unit hydraulic gradient in conditions of laminar flow using Darcy’s law. This parameter is valuable for assessing the material’s ability to waterproof the rest of the substructure.

**Figure 3:**
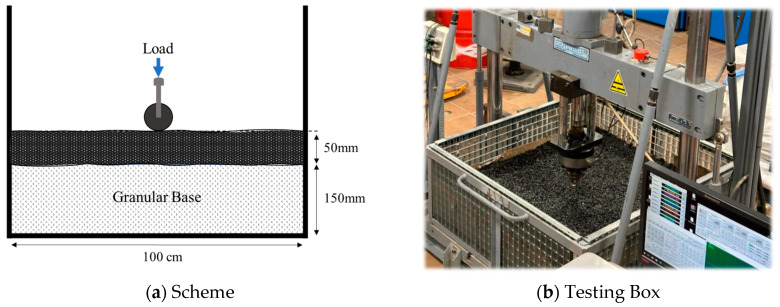
Full-scale testing box.

Finally, with the aim of simulating real operating and confinement conditions, a test box of 100 cm × 100 cm was constructed. The samples under study were layered with a thickness of 150 mm, compacted at the optimal moisture content, and in the case of RAP, re-compacted 24 h later at 60 °C using ultraviolet lights. A BBTM-11B asphalt mixture (UNE-EN 13108-2 [55]), produced with PMB 45/80-60 bitumen, with a thickness of 50 mm, was placed on the reference materials, as shown in Figure 3.

**Figure 4:**
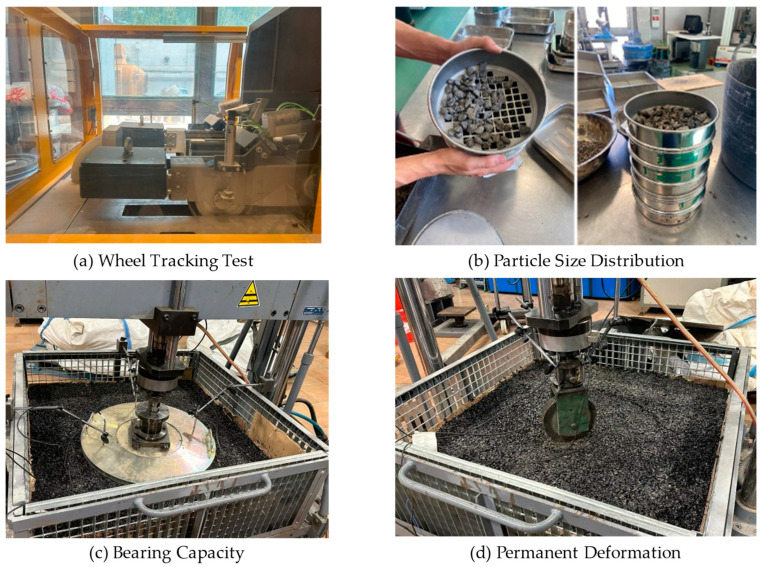
Setup of some of the conducted tests.

On the samples previously prepared in the test box were conducted load-bearing tests, using a 600 mm loading plate and under static and dynamic loads according to the standards UNE 103808:2006 [51] and UNE 103807-2 [52], respectively, using a 100 kN capacity loading frame, as described by Sol-Sánchez et al. [56]. For the first case, 2 cycles of load and unload were applied, with load levels of 35 kPa, 70 kPa, 105 kPa, 150 kPa, 175 kPa, 210 kPa, and 245 kPa. In the dynamic load test, load cycles of 180 kPa with a frequency of 5 Hz were used. Finally, permanent deformation tests were conducted by applying 110,000 load cycles with an amplitude of 700 kPa and a frequency of 5 Hz, using a wheel with a diameter of 20 cm and a thickness of 40 mm. Figure 4 shows some of the conducted tests.

## 3. Results and Discussion

### 3.1. Optimization Study of the Use of RAP as a Granular Layer

#### 3.1.1. Influence of RAP Type

**Figure 5:**
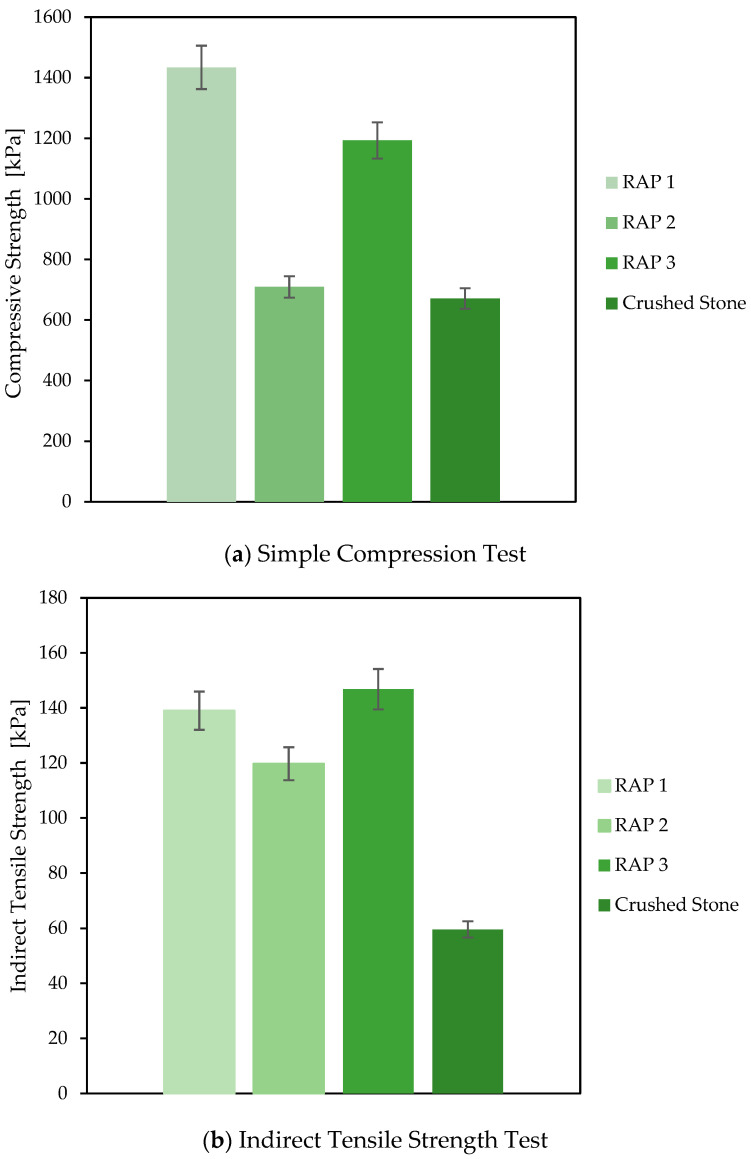
Indirect tensile strength and compression test results.

Figure 5 shows the results of (a) simple compression and (b) indirect tensile strength tests conducted in this study, comparing the strength of different types of RAP (compacted at 60 °C to provide the potential bonding benefits from residual asphalt binder in the recycled aggregates) in reference to natural crushed stones. The results indicate that the average compression strength of recycled materials was higher than that of the reference material, showing an average improvement of 65%. This aligns with findings reported by Alam et al. [57], who observed the competitive mechanical performance of RAP materials compacted at optimal moisture and ambient temperature under similar conditions, highlighting the potential of RAP to achieve comparable or superior performance to natural aggregates. In this case, RAP 1 and RAP 3, which have a more continuous gradation, exhibited the best performance, which could be related to a stringer mineral skeleton in comparison to RAP 2.

More significant improvements were observed in indirect tensile strength, with recycled materials outperforming reference materials by an average of 126%. This notable increase highlights the critical role of the bonding capacity provided by the residual bitumen in RAP, which contributes significantly to resistance under tensile stresses and increases the bearing capacity [19]. Similarly, RAP 1 and RAP 3 demonstrated superior performance, while RAP 2, with a more discontinuous gradation and lower asphalt content, exhibited inferior behavior in both compression and indirect tensile stresses. These findings indicate that the type of RAP plays a crucial role in enhancing the performance of sustainable granular layers, emphasizing the importance of carefully selecting RAP materials based on their gradation and asphalt content.

**Figure 6:**
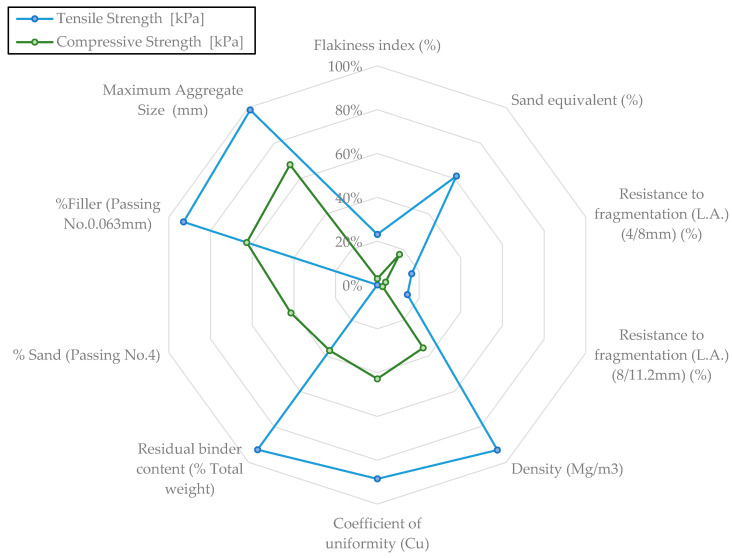
Laboratory materials correlations.

In light of the potential impact of RAP characteristics on its mechanical performance, correlations were performed between the physical properties of the materials (Table 1) and the measured compressive and tensile strengths in this part of the study. Figure 6 presents the coefficients of determination (R²) for each parameter. The farther away the points are from the center of the graph, the stronger the correlation. Both measured mechanical parameters show a significant relationship (R² > 60%) with the maximum aggregate size and filler content (% material passing the 0.0063 mm sieve).

The indirect tensile strength appears to correlate well with density, particle size distribution measured with the coefficient of uniformity (Cu), and the residual asphalt content of the recycled material, contributing to the overall adhesiveness and mechanical integrity of the materials [58,59]. On the other hand, the compressive strength is closely related to the maximum density obtained from the Modified Proctor test. This suggests that while aspects such as gradation can be optimized during material selection and mix design, other inherent properties of RAP, such as binder content and fine aggregate quality, also play a critical role and must be carefully considered to ensure structural performance.

#### 3.1.2. Impact of Combining RAP with Reference Materials

**Figure 7:**
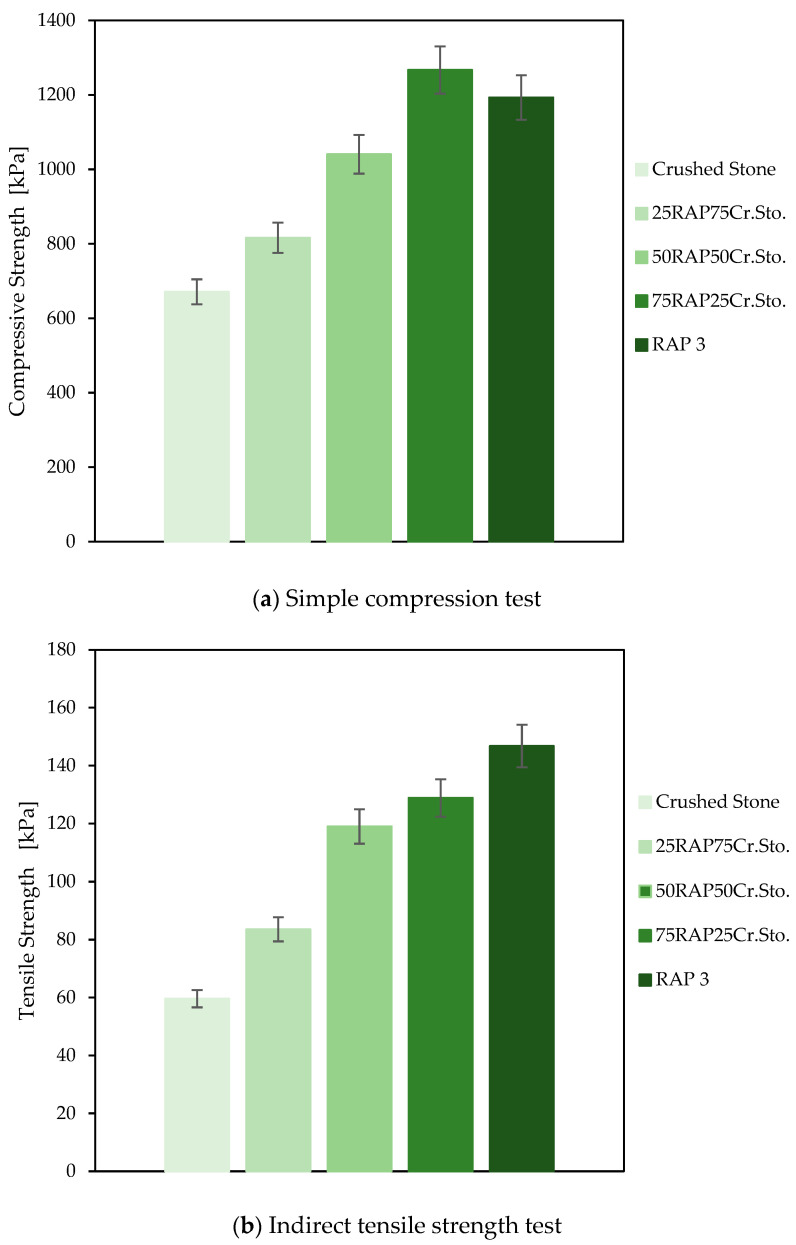
Impact of combining RAP with reference materials on mechanical behavior.

To analyze the effect of combining RAP with natural aggregates, as examined in previous studies [60,61]. Figure 7 shows the results of indirect tensile strength and simple compression tests for proposed replacements of 25%, 50%, and 75%, along with cases of 100% crushed stone and 100% RAP type 3 (best performed RAP). For this analysis, the results of combining RAP 3 are shown, as it exhibited a more continuous gradation, which is a key factor according to previous findings. The findings suggest that, in all cases, the mechanical performance of the crushed stone is enhanced with the incorporation of RAP, reaching conditions very similar to those of RAP (>80% of indirect tensile strength and simple compression) with replacements starting from 50% by weight of the recycled material.

However, it is important to note that some studies limit the use of RAP to proportions around 40–50% [62], finding reductions in properties such as CBR strength when proportions exceed 60% [3]. These limitations are primarily associated with excessive deformations observed at higher replacement levels [63]. Nevertheless, the contrast with the results reported in this study may be attributed to the fact that the material was compacted at its optimal moisture content and, subsequently, re-compacted at high ambient temperatures. Under these conditions, the residual bitumen in RAP can start to enhance cohesion, improving mechanical performance [19]. This aspect is crucial for future applications, highlighting the importance of compaction temperature as a key factor influencing the performance of RAP-based granular layers.

Moreover, Moreover, while high RAP content may raise concerns about compliance with standard engineering specifications, particularly regarding particle size distribution and shape indices, partial replacement strategies offer a viable solution. By adjusting the proportion of RAP in the mix, it is possible to balance mechanical performance while maintaining compliance with engineering standards. Previous studies [3,64] suggest that partial replacements of RAP can influence physical properties, such as increased resistance to LA abrasion, decreased absorptions, and maximum dry densities. These findings reinforce the potential of partial RAP incorporation as a strategy to mitigate the drawbacks associated with high RAP content, while still maximizing the sustainability benefits of recycled materials.

### 3.2. Bearing Capacity

**Figure 8:**
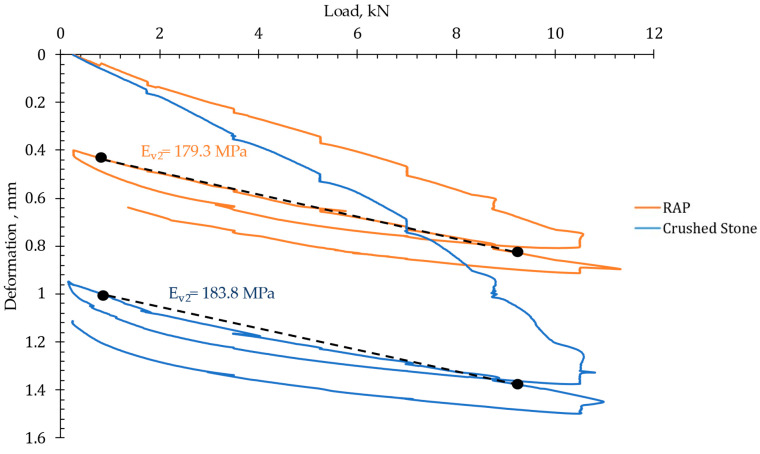
Static plate load test results.

Considering the previous outcomes, the subsequent analysis explores the mechanical behavior to its application as a base layer, with a particular emphasis on RAP 3 (considered the optimum RAP in this study). Figure 8 summarizes the results of the static plate load test, similar to that reported by other authors [65,66]. The results indicate that the use of RAP resulted in values comparable to those recorded by the reference material. In addition, it is crucial to note that the total deformation after the two loading cycles was lower for the recycled material compared to the reference material, which resulted in a lower modulus ratio (E_V2_/E_V1_), indicating a layer with superior mechanical performance in terms of load-bearing capacity. Therefore, these results suggest that layers with high RAP content do not necessarily lead to excessive deformations and reduced bearing capacity, as observed in previous studies [19,67]. When the residual bitumen is effectively utilized, RAP can provide significant mechanical advantages, enhancing cohesion and overall structural performance.

In summary, the static load plate test suggests that, despite being a layer composed of smaller-sized material with less continuity in its particle size distribution, the residual asphalt binder contributes to the resistance capacity. The static plate load results further underscore the feasibility of using RAP as a base layer material, particularly for applications where deformation and load-bearing capacity are critical.

**Figure 9:**
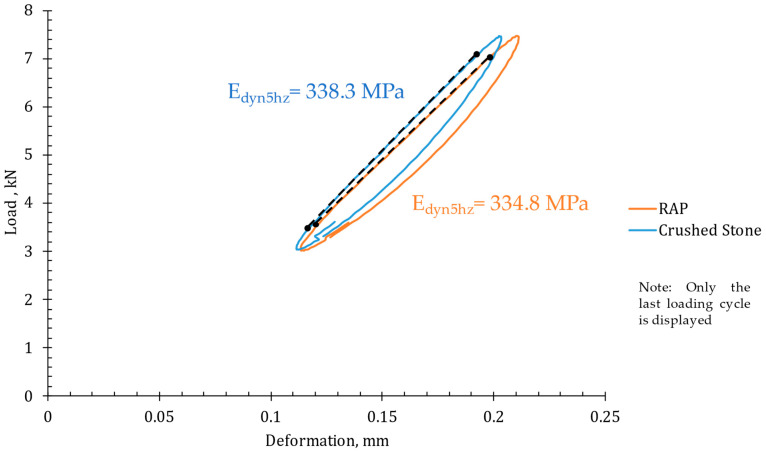
Dynamic plate load test results.

Similarly, Figure 9 presents the results of load-bearing capacity under dynamic loading (last cycle). In this case, the total deformation after 10 loading cycles is very similar for both materials. This finding is consistent with other studies reporting the suitability of RAP for dynamic applications when compacted at optimal moisture levels [62,63].

In terms of bearing capacity, the vertical elastic modulus (Edyn5hz) reached by the recycled materials is comparable to that of the material commonly used for these applications under this load condition, with minimal differences observed. The load-bearing capacity of the base layer is essential for effectively distributing stress to the underlying sublayers and minimizing settlement.

### 3.3. Permanent Deformation

**Figure 10:**
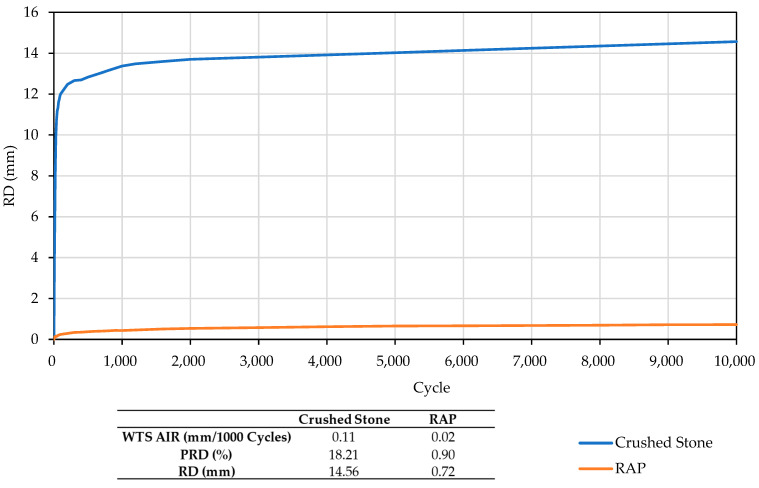
Wheel tracking test results.

The results of the wheel-tracking test, simulating the traveling of tire wheels over the layers of each material is presented in Figure 10, in this case, represents the evolution of layer deformation under the continuous passage of the wheel, presenting values of wheel-tracking slope (WTS, measured in millimeters of rut depth per 1000 wheel passes), mean proportional rut depth (PRD in %), and the depth of the rut (RD in mm). The results indicate that in this case, the recycled materials exhibit better performance than the reference material, suggesting that these recycled materials may experience lower total deformations during their application, Specifically, reductions of over 95% in the RD value were observed compared to the reference material.

However, some authors have reported higher rutting in RAP-based materials [68]. This discrepancy in findings may be attributed to the inherent heterogeneity of RAP, coupled with its physical properties, which play a critical role in its final performance. Factors such as bitumen content, gradation, and other physical characteristics significantly influence the material’s behavior under load application. The values obtained in this study, while higher than those reported by other authors [65], align with findings from other research that highlight the consistent behavior of RAP under controlled conditions [69,70]. Furthermore, the recycled material demonstrated stable performance throughout the test, contrasting with the reference material, which exhibited rapid settlement during the initial cycles before reaching stabilization.

### 3.4. Permeability

The constant head permeability test was conducted until the water flow through the material stabilized. In this case, the values of vertical permeability coefficients (K) were calculated for each of the studied materials, obtaining values of 9.76 × 10^−6^ m/s for the recycled material and 8.24 × 10^−6^ m/s for the reference material; however, values were considerably higher than those reported in other studies [71]. It is important to note that in those studies, the authors incorporated fillers, such as cement, at approximately 3% in unbound base layers, aiming to reduce voids and improve permeability coefficients.

Overall, it is observed that the recycled material has a slightly lower capacity to protect the lower layers of the substructure. However, all materials exhibited permeability coefficients suitable for their application. This parameter is closely related to gradation, so having continuous gradation contributes significantly to the material’s ability to protect the structure [72,73].

### 3.5. Full-Scale Laboratory Test Box

Based on the previous results, a full-scale test box was conducted to simulate operational and confinement conditions for the RAP and reference base layer. Tests were carried out on these materials to assess their load-bearing capacity and settlement under real conditions expected during the application of this solution.

#### 3.5.1. Bearing Capacity of Full-Scale Texting Box

**Figure 11:**
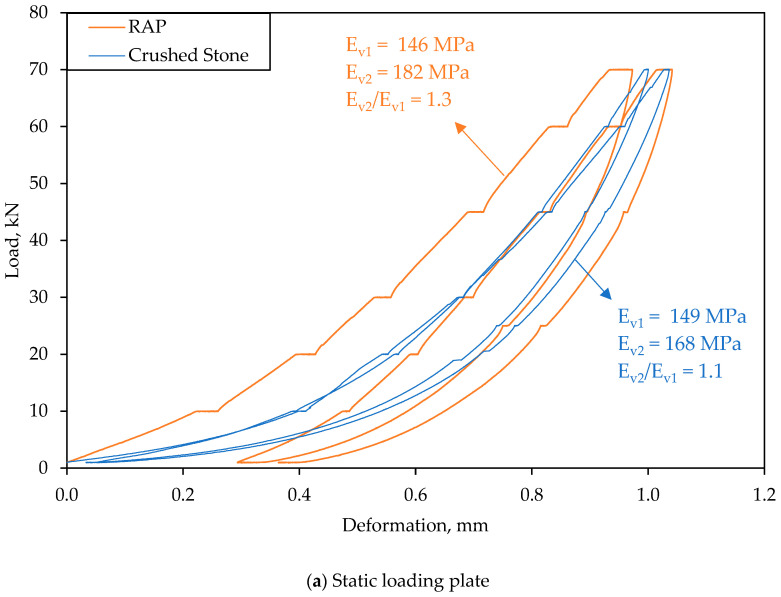

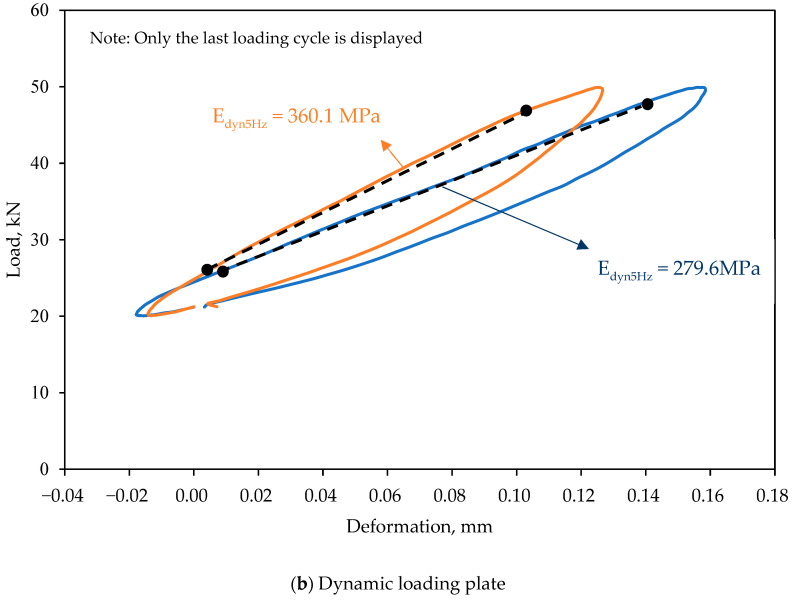
Bearing capacity.

Figure 11 shows the results of elastic modulus under (a) static load and (b) dynamic load for the materials under study. These indicate that the load-bearing capacity of the recycled materials was slightly higher than that of the reference material, both under dynamic and static loads. In Figure 11a, the values of the elastic modules for the first and second cycles in the static plate load test are presented, along with the relationship between them. Overall, the modulus of elasticity in the second cycle of load was about 8.3% higher in the recycled material than in the reference material. However, it can be observed that the relationship between the modulus of elasticity in the second cycle and that of the first cycle was better in the case of the reference material, although both were close to 1. The total deformation after both cycles is similar for both materials. The observed load-bearing capacity value is slightly lower than that reported in the previous section; however, it tends to be more realistic due to the closer approximation to in-service confinement conditions.

Moreover, the elasticity modulus values under dynamic loads are higher for the recycled material compared to the reference material, confirming better load-bearing capacity under these conditions. Furthermore, the total deformation observed in the reference material is greater than that of the recycled material, indicating a higher settlement tendency.

It is important to highlight that these findings are consistent with the results obtained at the specimen scale in previous sections, demonstrating the reproducibility of laboratory results when applied to full-scale test scenarios. This consistency further validates the suitability of RAP-based granular layers in infrastructure applications. Similar conclusions were drawn from previous full-scale test box studies, where the mechanical performance of RAP-based materials was assessed under real operational confinement conditions [74,75].

#### 3.5.2. Influence on the Behavior of the Track Section

**Figure 12:**
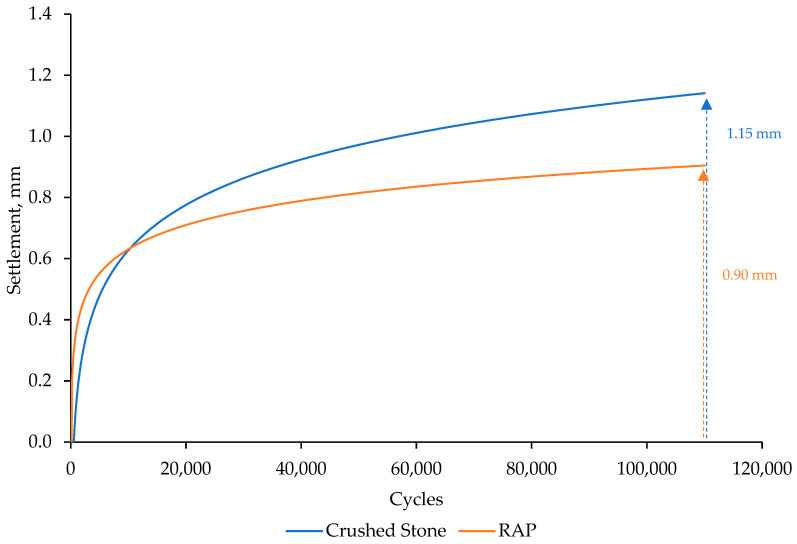
Permanent deformation.

Figure 12 illustrates the evolution of permanent deformation in each of the studied materials after 110,000 loading cycles. Under more realistic confinement conditions, the recycled material exhibited lower permanent deformations than those observed in the reference material, in this case, with a reduction of 22% in total permanent deformation. The results indicate that the deformation slope of the recycled material in the initial stage (first 500 cycles) was higher, but once consolidated, the deformation slope decreased, resulting in a smaller final deformation.

The observed reduction in total settlement highlights the importance of stiffer base layers, as an increase in substructure stiffness leads to decreased settlements and enhanced resistance over time for the entire track [76]. This translates into lower maintenance requirements and a reduction in infrastructure management costs. The improved performance of the recycled material under cyclic loading suggests that RAP-based layers can enhance long-term durability, minimizing permanent deformation and potentially extending the service life of the pavement with reduced maintenance interventions.

## 4. Conclusions

The main objective of this study is to analyze the feasibility of using reclaimed asphalt pavement (RAP) material as a granular base in road infrastructure projects using advanced laboratory tests and full-scale laboratory test boxes to simulate real conditions. Based on the results obtained from laboratory tests characterizing different RAP types and evaluating the influence of various design parameters, the following conclusions can be drawn:

- The recycled materials analyzed in this study exhibited appropriate mechanical characteristics to be used as aggregates in base layers for road structures.
- The results demonstrate that the use of RAP enhances the internal cohesion and mechanical resistance of granular materials used in road-based applications. This improvement is attributed to the bonding capacity provided by the residual binder, which becomes activated at high ambient temperatures. This approach resulted in a 65% increase in compressive strength and up to a 126% increase in indirect tensile strength compared to reference materials.
- The load-bearing capacity of RAP-based materials was superior to that of conventional granular bases under the proposed compaction conditions, achieving up to 30% higher modulus of elasticity below static and dynamic loads.
- Despite its more discontinuous gradation, RAP demonstrated adequate permeability characteristics, ensuring sufficient protection for the lower layers of the pavement structure against water infiltration.
- In permanent deformation tests, RAP-based materials exhibited superior performance, with up to a 22% reduction in total permanent deformation compared to natural aggregates, which translates into lower maintenance needs over the pavement’s service life.

Based on these conclusions, it has been demonstrated that RAP can be effectively used in high proportions as an unbound base layer, provided that appropriate compaction techniques are employed to facilitate the activation of the residual asphalt binder. Nonetheless, it is important to remark that although this study includes different RAP sources, it is difficult to establish universally applicable conclusions due to the heterogeneity of recycled RAP materials, as variations in aggregate type, binder properties, and asphalt content significantly influence their mechanical behavior.

Thus, further research is needed to assess the environmental and economic viability of RAP in granular-based applications. This includes comprehensive studies on life cycle assessments and cost analyses that consider potential reductions or increases in layer thickness to optimize performance and sustainability. A comparative evaluation of RAP reuse in different pavement layers, including asphalt mixtures and granular bases, is essential to determine the most sustainable and efficient application. Future studies should also focus on evaluating long-term field performance to validate laboratory findings and ensure practical implementation.

## Figures and Tables

**Table 1:** Materials’ main properties.

Properties	Standard	Crushed Stone	RAP 1	RAP 2	RAP 3
Flakiness index (%)	UNE EN 933-3:2023 [30]	18	13	6	11
Percentage of fractured face (%)	UNE-EN 933-5:2023 [31]	100	100	100	100
Sand equivalent (%)	UNE-EN 933-8:2015 [32]	55	78	81	69
Resistance to fragmentation (L.A.) (4/8 mm) (%)	UNE EN 1097-2:2021 [33]	23.9	20.7	15.3	20.8
Resistance to fragmentation (L.A.) (8/11.2 mm) (%)	UNE EN 1097-2:2021 [33]	20.8	17.1	12.8	18.7
Density (Mg/m^3^)	UNE-EN 1097-6 [34]	2.81	2.57	2.57	2.56
Coefficient of uniformity (Cu)	UNE-EN 933-1:2012 [35]	45	8	10	11
Curvature coefficient (Cc)	UNE-EN 933-1:2012 [35]	3.1	3.1	3.1	1.4
Modified Proctor Opt. Moisture content (%)/Max Dry Density. (g/cm^3^)	UNE 103501:1994 [36]	5.0/2.37	5.5/2.25	5.5/2.32	5.5/2.33
Recovery of binder by Evaporation					
Residual binder content (% Total weight)	UNE-EN 12697-1:2013 [37]	-	3.9	3.0	4.0
Binder Penetration (25 °C) (0.01 mm)	UNE-EN 1426:2015 [38]	-	16.0	4.0	8.0
Softening Point (°C)	UNE-EN1427:2015 [39]	-	71	129	85

**Table 2:** Testing plan.

Stage	Properties	Materials	Test
Optimization Study of the Use of RAP as a Granular Layer	Impact of RAP Type Impact of Combining RAP with Reference Materials	Crushed Stone RAP 1 RAP 2 RAP 3	Indirect Tensile Strength Compressive Strength
Bearing Capacity	Elastic Modulus	Crushed Stone Opt. RAP	300 mm Static Plate Bearing Test 300 mm Dynamic Punching Test
Permanent Deformation	Total Rut Depth		Wheel Tracking Test
Permeability	Vertical Permeability (K)		Permeameter
Full Scale Laboratory Testing Box	Bearing CapacityPermanent Deformation		600 mm Static Plate Bearing Test 600 mm Dynamic Punching Test Permanent Deformation

## Data Availability

The data that supports the findings of this study are available from the author upon reasonable request.
